# Data Acquisition System for On-the-Go Soil Resistance Force Sensor Using Soil Cutting Blades

**DOI:** 10.3390/s22145301

**Published:** 2022-07-15

**Authors:** Vladimír Cviklovič, Miroslav Mojžiš, Radoslav Majdan, Katarína Kollárová, Zdenko Tkáč, Rudolf Abrahám, Soňa Masarovičová

**Affiliations:** 1 Institute of Electrical Engineering, Automation, Informatics and Physics, Faculty of Engineering, Slovak University of Agriculture in Nitra, 949 76 Nitra, Slovakia; vladimir.cviklovic@uniag.sk; 2 Institute of Agricultural Engineering, Transport and Bioenergetics, Faculty of Engineering, Slovak University of Agriculture in Nitra, 949 76 Nitra, Slovakia; miroslav.mojzis@uniag.sk (M.M.); radoslav.majdan@uniag.sk (R.M.); zdenko.tkac@uniag.sk (Z.T.); rudolf.abraham@uniag.sk (R.A.); 3 Information and Coordination Centre of Research, Faculty of Engineering, Slovak University of Agriculture in Nitra, 949 76 Nitra, Slovakia; 4 Department of Geotechnics, Faculty of Civil Engineering, University of Žilina, 010 26 Žilina, Slovakia; masarovicova@uniza.sk

**Keywords:** load cell, datalogger, microcontroller, force, mechanical resistance, soil compaction

## Abstract

Worldwide, agricultural land is a dominant part of the environment. It is very important to understand the physical properties of soil because they directly or indirectly affect the entire human population. This paper proposes a data acquisition system for an original design of the soil resistance force sensor (SRFS). It serves to evaluate the properties of soil affected and unaffected by tractor passages through the field. The SRSF uses two cutting blades to measure soil mechanical resistance within the tire track and outside the tire track. The proposed system consists of two load cells, datalogger, power supply and software for personal computers. The system was practically tested under field operation. The results showed significant differences between the soil resistance force measured outside the tire track and within the tire track after one, two and three tractor passages. The data were compared with penetrometer resistance and soil bulk density, standardly characterizing soil mechanical resistance. An increase of soil resistance force after one, two and three tractor passages corresponded with an increase in reference parameters. The results showed that the proposed system is suitable for practical applications to evaluate soil mechanical resistance using SRFS.

## 1. Introduction

In general terms, a data acquisition system consists of software and hardware to measure a physical characteristic of certain natural phenomena. In many cases, physical characteristics are properly transformed by sensors to allow conversion into digital numeric values to be manipulated by a computer. Considering an agricultural sector, various sensors in contact with soil are often used to obtain a signal for subsequent processing. The sensors of various design concepts using one of the following measurement methods have been developed:

- Electrical and electromagnetic sensors measure electrical resistivity/conductivity, capacitance or inductance affected by the composition of tested soil;
- Optical and radiometric sensors use electromagnetic waves to detect the level of energy absorbed/reflected by soil particles;
- Mechanical sensors measure forces resulting from a tool engaged with the soil;
- Acoustic sensors quantify the sound produced by a tool interacting with the soil;
- Pneumatic sensors assess the ability to inject air into the soil;
- Electrochemical sensors use ion-selective membranes that produce a voltage output in response to the activity of selected ions (H^+^, K^+^, NO3^−^, Na^+^, etc.) [1].

The sensors mentioned above were mainly designed in response to the development of advanced technologies and precision agriculture. Precision farming manages fields non-uniformly with consideration of spatial variation in comparison with conventional farming. The spatial variation of soil properties is characterized in terms of the effect on crop yield [2]. For this purpose, on-the-go sensor types were designed and manufactured [3,4]. These sensors allow assessment of the soil profile in real time in contrast to the traditional method of exploring within-field soil variation through grid sampling. The principle requires determination of soil properties by laboratory testing of collected samples. The need for information about fields makes the laboratory tests time-, cost-, and labor-consuming [5]. Some sensors use the stop-and-go principle, which does not require laboratory testing of soil samples. The advantage of this principle is that the information about the measured parameters is immediately available, but the main disadvantage is a discontinuous mode of operation [6]. 

Besides the positive effects of agriculture intensification, a deterioration in the physical properties of soil due to soil compaction is increasing at present. The evaluation of the physical characteristics of soil is very important for agriculture as well as for the global environment. Soil compaction negatively affects the environment and human population because it reduces soil fertility and water infiltration [7]. The information on soil compaction is also used to evaluate the traction needed for agrotechnical operations [8,9,10] or for optimalization of tillage parameters in precision agriculture. The movement of agricultural machinery has a negative impact on the soil because its weight acts on the soil through wheels or tracks. The increase in machinery weight reduces wheel slip and improves tractive efficiency. Hence, a powerful engine requires an adequate weight on axles. On the other hand, an increase in machinery weight and movement intensity through the field causes an increase in soil compaction [11,12,13] characterized by various parameters—for example, soil bulk density, penetrometer resistance, porosity, etc. [14]. 

Because soil compaction is a serious worldwide problem, various devices based on the measurement of soil mechanical resistance were developed and applied in practical conditions. The vertical and horizontal measurement systems are the two base principles. The vertical principle is suitable also for hand portable devices in contrast with the horizontal principle. Both systems can be mounted on the tractor or another vehicle to evaluate a large area, but a certain disadvantage of the vertical systems [15,16,17] is the stop-and-go operation’s providing point measurements. On the other hand, the horizontal systems allow on-the-go measurement and application during agrotechnical operations to reduce tractor passages and cost for measurement. Considering that the soil has been deposited in a layer-wise manner, the vertical and horizontal systems differ in forces acting on the sensor. The methodology using the vertical cone penetrometer is standardized and is a reference for horizontal systems. 

**Figure 1:**
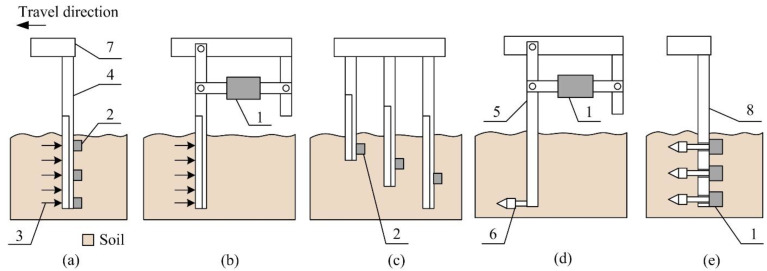
Location of various sensors for measurement of soil mechanical resistance depending on the device design: (**a**) Strain gauges on the cutting blade; (**b**) Load cell connected with the cutting blade; (**c**) Multiple strain gauges on cutting blades; (**d**) Load cell and lever with penetrating cone; (**e**) Multiple load cells with penetrating cones: 1—load cell; 2—strain gauge; 3—soil resistance force; 4—cutting blade; 5—force lever; 6—penetrating cone; 7—frame; 8—shin.

The basic part of all horizontal systems is a segment moving in the horizontal direction to penetrate or cut the soil in a certain depth (Figure 1). In most cases, the load cell or strain gauges are used to convert soil resistance into an electric signal. To evaluate this parameter in various depths, a cutting blade can be equipped with multiple strain gauges [18], or multiple cutting blades with various lengths can be used [19,20]. The sensors of some devices are mechanically connected with a penetrating cone or prismatic tip [21]. This design allows the use of more load cells with cones [22,23,24] to evaluate soil compaction in various soil depths. Sensors using one vertical blade equipped with several strain gauges were developed to evaluate soil properties at various depths. The vertical blade must be designed to eliminate deformation and bending moments, which are detected by strain gauges instead of soil strength. Hence, the idea of discrete blade for each depth with the aim of eliminating the interaction between the main blade and sensing tip was developed [25]. Each of the four instrumented shanks consisted of an extended octagonal load cell. This system showed lower variance of measured values in comparison with a handheld cone penetrometer. A similar sensor design uses a multiple blade measurement system with three independent blades [26]. Using the penetrating cone, soil mechanical properties can be expressed by penetrometer resistance [27]. The research showed that a prismatic tip is a better solution than a conical tip because it generates data that better correlate with the cone penetrometer. The conical tip is loaded in all directions, but the prismatic tip is loaded in sideway directions only [28]. Soil moisture is a parameter that significantly affects the soil’s physical properties. The soil moisture sensor integrated into the penetrating cone allows the simultaneous measurement of soil moisture content and soil resistance [29,30,31]. The location and type of sensors depends on the design of the measuring device, as mentioned above. The devices of simpler principles use the segment in contact with the soil, and the load cell is loaded through a lever mechanism. The load cell can be located outside the soil and protected against the moisture, stones, and other negative factors. Multiple sensors located in the soil profile are used to measure soil mechanical resistance in various soil depths. 

This study presents the on-the-go measurement system of soil mechanical resistance. The data on the mechanical properties of the soil affected and unaffected by tractor passage are significant for evaluation of tractors’ influence on the soil due to soil compaction. The design of the soil resistance force sensor (SRFS) is based on previous studies and uses two cutting blades in contact with the soil. The innovation of the device is based on the measurement of two parameters. Soil resistance force is evaluated within the tire track and outside the tire track (crop bed) at the same time. It allows the evaluation of soil properties after tractor passages through the field. Agricultural production improvement requires knowing the effect of the tractor on the soil with specific properties resulting from agrotechnical operation, actual soil moisture and soil type. It is important to characterize the influence of tractor parameters as various tractor wheel types equipped with various tires, tractor load and its distribution on axles, driving wheels slip, drawbar pull, etc., on soil properties. Considering the specific design of SRFS, a data acquisition system with some specific features was proposed for practical application. The aim of this study was (1) to present the original design of SRSF, (2) to present the data acquisition system consisting of two load cells, power supply and datalogger, allowing data transfer to the computer via software, and (3) to test the SRSF with the proposed data acquisition system based on soil resistance force measured outside the tire track and within the tire track after one, two and three tractor passages. 

## 2. Materials and Methods

### 2.1. Design of Soil Resistance Force Sensor

**Figure 2:**
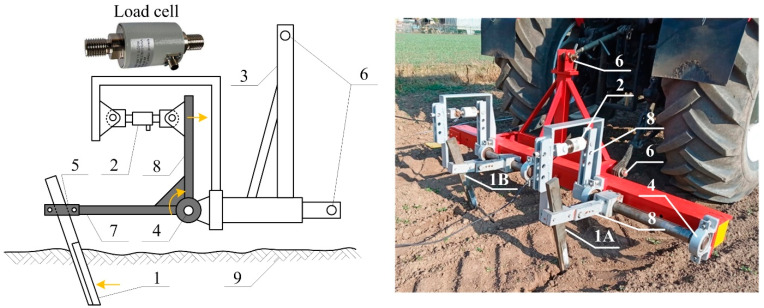
Scheme and design of SRSF (yellow arrows mean sensor parts motion during operation): 1—cutting blade; 1A—cutting blade within the tire track; 1B—cutting blade outside the tire track; 2—load cell; 3—frame; 4—pivot; 5—cutting blade fastening; 6—connection to the three-point hitch; 7—horizontal lever; 8—vertical lever; 9—soil.

The device for on-the-go measurement of soil resistance force was developed at the Institute of Agricultural Engineering, Transport and Bioenergetics of the Faculty of Engineering, Slovak University of Agriculture in Nitra (Figure 2). The design of SRFS uses two cutting blades with load cells located outside the soil. It continuously measures two parameters by each cutting blade. The first parameter characterizes the soil mechanical properties before the negative impact of the machine wheel on the soil and the second one after that. This design allows the evaluation of the mean value of soil mechanical resistance in the soil profile. The device can be used for evaluation of soil compaction or for determining the traction required for soil tillage tools. The device is attached to the tractor’s three-point hitch (6). Two cutting blades (1) to cut through the soil are the main parts of the device. One cutting blade (1A) is placed behind the tractor’s rear wheel to characterize the soil compacted by tractor weight. The second cutting blade (1B) characterizes the soil not compacted due to tractor passage. The cutting blades with the total length of 600 mm were made of steel bar with a rectangular cross-section (50 mm × 20 mm). A bottom sharpened section with a length of 300 mm was designed for contact with the soil. The cutting blades were inclined at 30° to:

- reach the working depth in all soil types (various physical properties) without the need for additional ballast weight;
- cut the roots of plants grown in the field.

The resistance of soil acts on the blades which rotate the horizontal and vertical levers (7, 8) around the pivot (4), as shown by orange arrows. Cutting blade fastening (5) allows the setting of various contact lengths of the blade with the soil and moments acting around the pivot. The vertical levers act on the load cells (2) which generate an electric signal depending on soil resistance force. The design allows the setting of one cutting blade within the tire track and another one outside the tire track for various tire tread widths of tractors. The analysis of the device principle was published by [32]. 

Two load cells EMS 150 (Emsyst, s.r.o., Trenčín, Slovak Republic) with a strain–gauge bridge (Wheatstone bridge) in a steel housing (accuracy class: 0.2, rated capacity: 10 kN, maximum non-linearity and hysteresis: 0.15% of full scale) were used in the device. This load cell is of a cylindrical industrial type for heavy-duty operation conditions. The body of the sensor is made of steel. The cylinder diameter is 80 mm, and the total length is 150 mm. The recommended excitation is 7–15 V.

The SRFS interacts with the soil during the measurements. Reece [33] analyzed a flat blade moving through the soil to define the universal earth moving equation. It characterizes soil deformation in contact with the tool. Based on this research, the resistance force acting on the cutting blade is described by the following equation:

(1) $$F=f\left(c,\;\gamma ,\;q,\;c_{a},\;\phi ,\;\delta ,\;b,\;\theta \right)$$

where *F*—resistance force acting on the blade (N); *c*—soil cohesion (Pa); *γ*—soil density (kg/m^3^); *q*—surcharge pressure (Pa); *c_a_*—soil-to-metal adhesion (Pa); *ϕ*—angle of internal shearing resistance (°); *δ*—angle of soil-to-metal friction (°); *b*—tool depth (m); and *θ*—rake angle of cutting blade (°). 

Therefore, the force required to deform the soil (blade moving through the soil) depends on the gravitational effect of soil, cohesive effect of soil and surcharge effect acting on the soil. Besides the soil properties mentioned above, resistance force also depends on blade (tool) geometry and blade-to-soil strength properties. Blade geometry factors are the angle of blade from horizontal plane (rake angle), curvature of blade shape and depth-to-width ratio.

**Figure 3:**
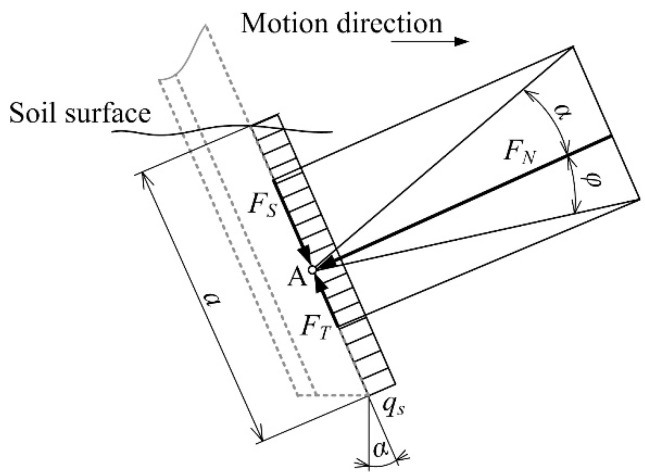
Determination of cutting blade inclination angle: *F_N_*—normal force; *F_T_*—frictional (tangential) force; *F_S_*—sinkage force; *q_s_*—specific load; *α*—cutting blade inclination angle; *φ*—frictional angle; *a*—contact length of cutting blade with soil; A—point of action.

The design of SRFS consisting of vertical and horizontal levers was proposed to eliminate the lifting forces acting on the cutting blades and resulting from the horizontal motion direction. A sinkage of the cutting blade to the soil depends on inclination angle *α* which affects the value of sinkage force *F_S_* as follows (Figure 3):

(2) $$F_{T}=q_{S}\;\cdot a\;\cdot \;\mathrm{tg}\varphi$$

The frictional force *F_S_* acts in the opposite direction to the sinkage force and causes lifting of the cutting blade, as follows (Figure 3):

(3) $$F_{S}=q_{S}\;\cdot a\;\cdot \;\mathrm{tg}\alpha$$

The normal force *F_N_* acting on the cutting blade equals the specific load *q_s_* multiplied by the contact length *a* of the cutting blade with the soil. To ensure the cutting blade sinkage to the soil, the sinkage force (cutting blade inclination angle) must be higher than the frictional force (frictional angle), as follows:

(4) $$F_{T}<F_{S}$$

(5) $$q_{S}\;\cdot a\;\cdot \;\mathrm{tg}\varphi <q_{S}\;\cdot a\;\cdot \;\mathrm{tg}\alpha$$

(6) φ<α

The cutting blade inclination angle’s being higher than the frictional angle ensures the sensor sinkage when it is in contact with the soil. For the chernozem soil, the cutting blade inclination angle of 30° was experimentally determined. The angle should be lower for loose sandy soil types. On the other hand, the angle should be higher for clay soil types.

**Figure 4:**
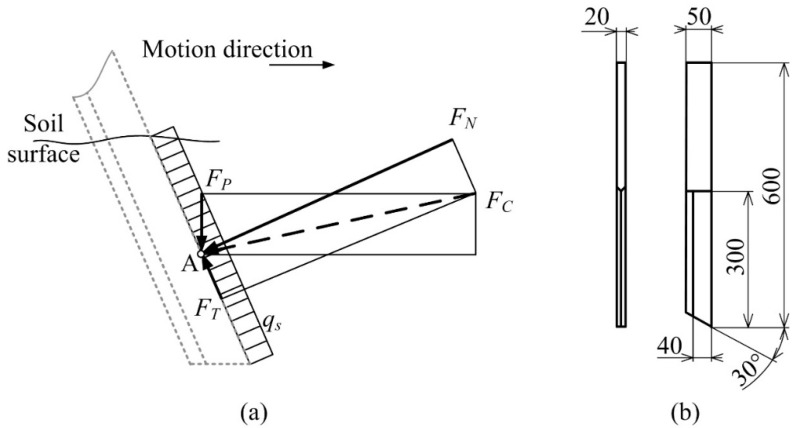
Cutting blade: (**a**) Forces acting on cutting edge: *F_N_*—normal force; *F_T_*—frictional (tangential) force; *F_C_*—soil cutting force; *F_P_*—perpendicular force; *q_s_*—specific load; *α*—inclination angle; A—point of action; (**b**) Dimensions.

The cutting force (*F_c_*) originates from the normal force (*F_N_*) and frictional force (*F_F_*) when the cutting blade is displaced forward (Figure 4a) [34]. The point of action of normal force is in the center of specific load (*q_s_*) distributed along the cutting edge, depending on the working depth. The horizontal force (*F_H_*) is calculated from the cutting force and perpendicular force (*F_P_*), with effort required to sink the cutting blade down into the soil due to inclination angle (*α*). The forces mentioned above depend on soil mechanical properties that are different for each soil type and moisture content. The dimensions of the cutting blade are shown in Figure 4b. 

**Figure 5:**
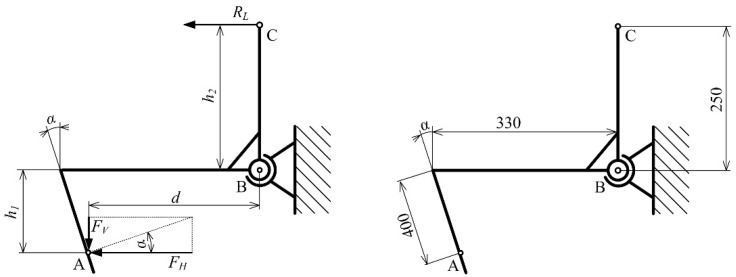
Forces acting on the SRFS and dimensions: A—point of action of forces acting on the cutting blade; B—support point; C—point of action of reaction acting on the load cell; *F_H_*—horizontal force; *F_V_*—vertical force; *R_L_*—load cell reaction force; *d, h_1_, h_2_*—specific distances; *α*—inclination angle.

Considering the forces acting on the cutting blade, the horizontal force (*F_H_*) can be expressed to characterize soil mechanical resistance [20]. The horizontal force is calculated from the moments acting around the support pivot (B) as follows (Figure 5):

(7) $$\sum^{\;}M_{B}=0:\;F_{H}\;\cdot h_{1}-F_{V}\;\cdot d-R_{L}\;\cdot h_{2}=0$$

(8) $$F_{H}\;\cdot h_{1}-\mathrm{tg}\alpha \;\cdot F_{H}\;\cdot \;d=R_{L}\;\cdot h_{2}$$

(9) $$F_{H}\;\cdot \;\left(h_{1}-\mathrm{tg}\alpha \;\cdot \;d\right)=R_{L}\;\cdot h_{2}$$

(10) $$F_{H}=R_{L}\;\cdot \;\frac{\left(h_{1}-\mathrm{tg}\alpha \;\cdot \;d\right)\;}{h_{2}}=R_{L}\;\cdot \;k$$

where *F_H_*—horizontal force (N); *R_L_*—load cell reaction force (N); *F_V_*—vertical force (N); *d, h_1_, h_2_*—perpendicular distances (mm); α—inclination angle (°); *k*—conversion factor. 

The SRFS allows the setting of equilibrium between the horizontal and load cell reaction forces when the conversion factor (*k*) is 1, changing the distance *h_1_* according to the measurement depth. The conversion factor *k =* 1.02 ≈ 1 was calculated (Figure 5) using the actual perpendicular distances to the point B (*h_1_* = 346 mm, *h_2_* = 250 mm and *d =* 157 mm). Similarly, when the maximum length of the cutting blade (600 mm) is used, and the full length of the cutting edge (300 mm) is sunk into the soil (*h_1_* = 373 mm, *h_2_* = 250 mm and *d =* 144 mm), the conversion factor is also 1. In the case of another cutting blade setting, the conversion factor must be used to calculate the correct value of force. 

### 2.2. Proposal of Data Acquisition System

**Figure 6:**
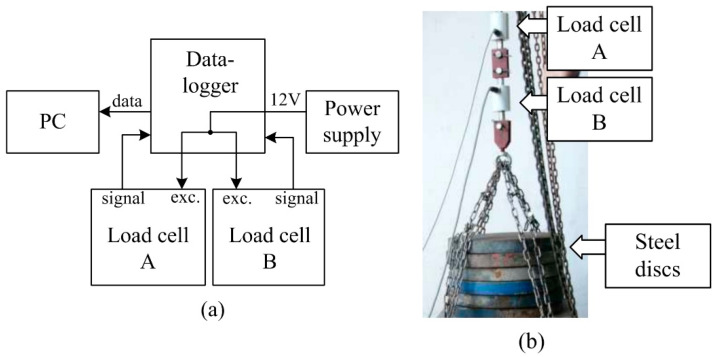
Measurement system: (**a**) Scheme of the system; (**b**) Calibration test using the predicted load.

The application of the SRFS requires the recording of the measured parameter. The datalogger, as the main part of the system (Figure 6), was proposed for this purpose. The datalogger must be proposed to separately record signals from two load cells in real time. The output signal from the load cells is proportional to load acting on the cutting blades. The sampling frequency of 10 Hz was proposed considering the relatively low operation speed. Low speed is required because of the dynamic forces elimination. The sampling frequency of 10 Hz was verified based on an experiment in which we evaluated the insignificance of components of frequencies higher than 5 Hz using FFT (Fast Fourier transformation). To excite the load cells, the direct voltage of 12 V was used from a charging power supply. The special software was required to transfer the data from the datalogger to the personal computer. The calibration test was performed to determine the deviation between the predicted load acting on the load cells and output forces recorded in the datalogger. 

### 2.3. Experimental Test under Field Conditions

**Figure 7:**
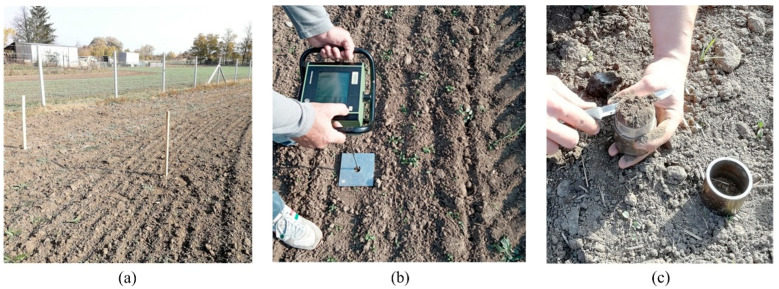
Experimental field: (**a**) Area bounded by plot stakes; (**b**) Measurement of penetrometer resistance outside the tire track using the handheld penetrologger; (**c**) Soil sampling by Kopecky’s soil core samplers.

Field experiments were used to test the functionality of the measurement system. When the SRFS with the data acquisition system was prepared, the measurements were performed in the autumn after wheat harvest. The field was cultivated by a disc harrow. Ten soil samples were randomly collected and analyzed. The value of soil moisture was 19.8% (dry basis) [35]. The soil was classified as Chernozem (World Reference Base for Soil Resources). The field is located in the district of Levice in western Slovakia (Figure 7a).

Three areas were bounded by plot stakes in the experimental field. The first area was cultivated soil without tractor passage. The second area contained compacted soil within tire tracks after one tractor passage. The third area included compacted soil within tire tracks after two tractor passages. During the measurements in the second and third area, the tractor moved in tire tracks. The data from the SRSF were compared with the soil compaction of all areas characterized by penetrometer resistance and soil bulk density within the tire track and outside the tire track [36] where cutting blades are placed. Each measurement was repeated three times, and average values were calculated. Penetrometer resistance was evaluated in ten soil depths. A handheld penetrologger Eijkelkamp (Eijkelkamp Soil & Water, Giesbeek, Netherlands) with a cone diameter of 10 mm and angle of 60° was used (Figure 7b). Soil bulk density was evaluated [7,37,38] from dry soil weight [39]. Kopecky’s soil core samplers with a volume of 100 mL were used to collect the soil samples, Figure 7c. The data experimentally measured were statistically evaluated using the GraphPad software. 

The SRFS was attached to the tractor Zetor 7245 equipped with the front tires 11.2–24 TZ19 (Barum, a. s., Otrokovice, Czech Republic) and rear tires 16.9–30 TZ13 (Barum, a. s., Otrokovice, Czech Republic). The velocity of the tractor was 2 km·h^−1^. The cutting blades cut the soil in the depth of 10 cm ± 2 cm (depth of soil cultivation by the disk harrow was 15 cm). The depth range from 8 to 12 cm was caused by the typical non-ideal level of the field after soil tillage. Soil resistance was continuously measured within the tire track and outside the tire track. Soil resistance and time were recorded by the proposed system.

## 3. Results

### 3.1. Design of Data Acquisition System

**Figure 8:**
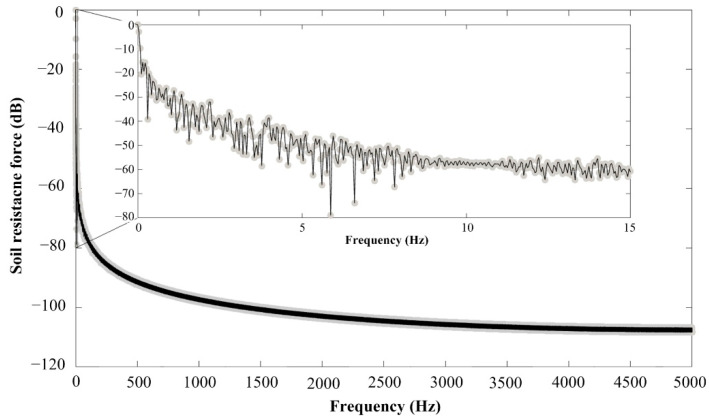
Single-sided amplitude spectrum of soil resistance force.

Output voltages from sensors are connected to the analogue input of the datalogger. Signals are filtered by active anti-aliasing filters of the fourth order described by Butterworth approximation. The cut-off frequency is set to 100 Hz. Anti-aliasing filters are low-pass in Sallen–Key connection. Due to the problematic suppression of signals above the frequency range of the operational amplifier, the filter is supplemented by a passive RC filter element with a cut-off frequency of 280 Hz. Subsequently, the signal is converted to digital form via a 24-bit AD converter. The final sampling frequency was set to 10 Hz because the measured data were averaged. The control algorithm contains oversampling to improve the measurement precision. Then, 95% of the absolute value of measured force was formed by frequency components below 5 Hz, so the sampling frequency of 10 Hz is adequate for data recording, Figure 8. 

**Figure 9:**
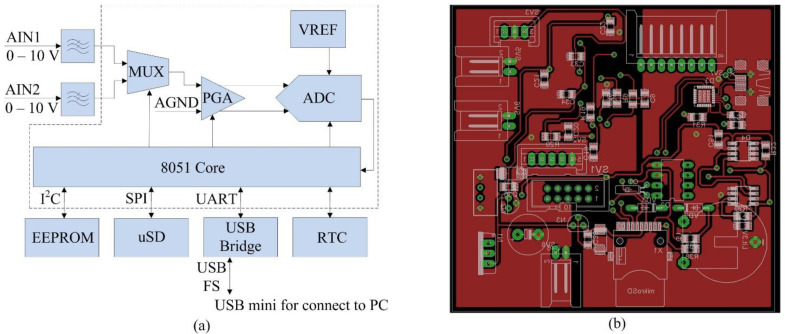
Datalogger: (**a**) Simplified scheme; (**b**) Printed circuit board.

A timestamp is assigned to each measured sample. The timestamp is read from the RTC circuit via I2C interface. The measurement process is controlled by the microcontroller C8051F350. (Silicon Labs Inc., Austin, USA) It uses a high-speed 8051 core with a number of peripherals. In our case, a 24-bit AD converter with an internal voltage reference, a programmable gain amplifier and UART, I2C and SPI interfaces are used. The measured data are stored in the EEPOM memories via the I2C interface due to the high reliability over a wide temperature range. The time-stamped data from the EEPROM are then read into the PC via the CP2102 gateway by the control application. USB FS is used for ease of connection. The structure of the hardware is shown in Figure 9.

The parameters of selected parts are described as follows:

- The AD converter is characterized by the voltage of 2.45 V at 25 °C, temperature coefficient of 15 ppm/°C, power supply rejection of 50 dB, resolution of 24 Bit, output word rate of 200 Hz, typical RMS noise of 76 µV, integral nonlinearity lower than ±15 ppm (FS) and gain error of ±0.002%.
- The microcontroller is characterized by the maximum rate lower than 50 MIPS, temperature range from −40 to +85 °C, program memory flash of 8 kB and maximum clock frequency of 50 MHz.

The firmware of the device was coded in a uVision application with the KEIL compiler in C programming language. Communication uses UART in connection with the USB communication gateway CP2101. The RS232 COM port is available on the software side. The advantage of the concept is the device’s own memory because it is not necessary to have a computer or a wireless communication interface available during the measurement itself. Communication with the software takes place at speed 115,200 Bd in setting 8-N-1 without flow control. Communication sentences are all American Standard Code for Information Interchange (ASCII). Calibration was performed with TTi1604; (Thurlby Thandar Instruments Ltd., Huntingdon, United Kingdom) accuracy and resolution are 0.08% and 10 µV, respectively. 

**Figure 10:**
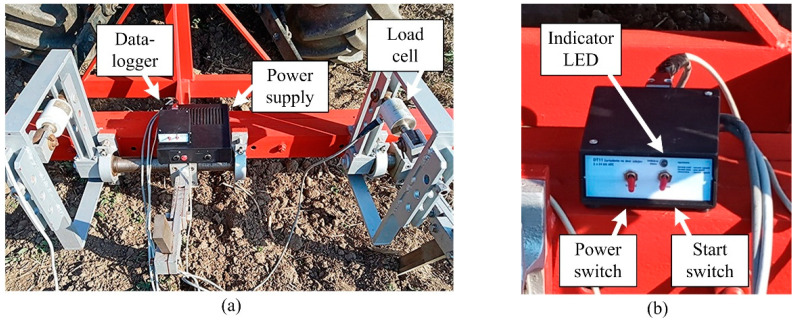
Datalogger: (**a**) Datalogger connected in the measurement system; (**b**) Controls and signalization.

The datalogger with power supply proposed for the SRFS is shown in Figure 10. The left switch is a power switch. The recording process is activated by the right start switch. To signalize an actual regime of operation, the datalogger was equipped with an LED indicator. The indicator constantly glowing red signalizes that the datalogger is connected to the power supply, but the recording process does not run. When the datalogger records the signals from the load cells, the indicator flashes green. When the memory is full, the indicator flashes red. 

Various designs of special devices for evaluation of soil mechanical resistance require the development of original measurement systems [40,41,42]. Some designs use only an A/D converter in combination with a personal computer, while other designs are equipped with standard or special dataloggers. Measurement under operation conditions often requires adopting the measurement system for the tractor. In the case of the device presented in this paper, the measurement system was developed for application in practical conditions. The initial measurement system used the universal portable recording device HMG 3010 (Hydac GmbH., Sultzbach, Germany). This instrument is relatively expensive and not designed for continual tractor operation under field conditions. It was necessary to set several recording options before use. It was used for the initial test of the device [20]. A new datalogger was developed to make the measurement easier and to increase the operation reliability. The proposed datalogger is operated by only two switches and compact design is suitable for the tractor cabin. A certain disadvantage is in the use of an external power supply. This problem can easily be solved using an electric system of the tractor to excite the load cells. 

**Figure 11:**
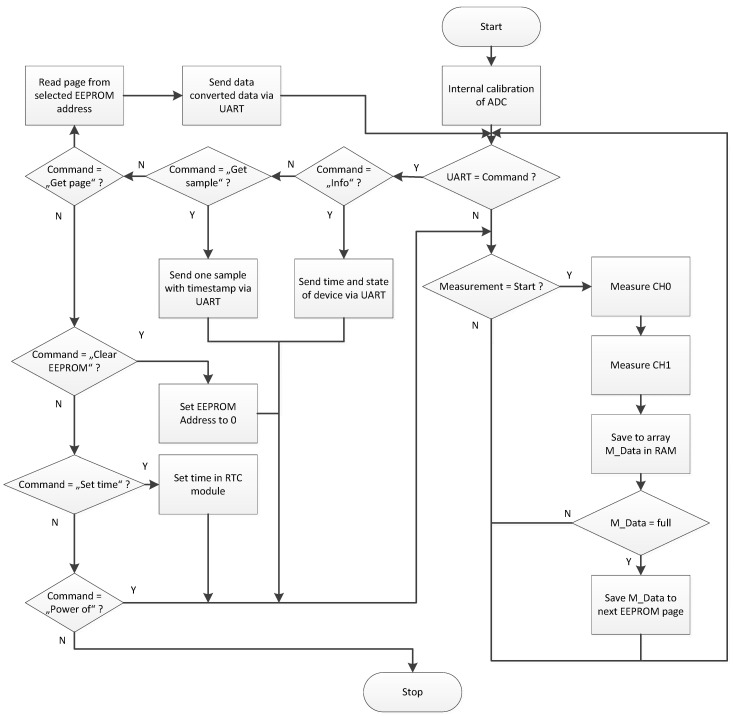
Flow chart of the proposed software.

The software was proposed to provide an interface through which the user can save, show, or visualize the data measured during the experiments. The software was written in the C# language in the MS Visual Studio Community application for control and data loading from the described hardware, (Figure 11). The NET component SerialPort for communication with hardware was used. The application consists of several threads for efficiency and controllability, even when downloading the measurements. Communication works on special tracks that are triggered as needed. The download speed of measurements is thus given by the maximum throughput of the communication interface. Control is solved through the MainMenu component.

**Figure 12:**
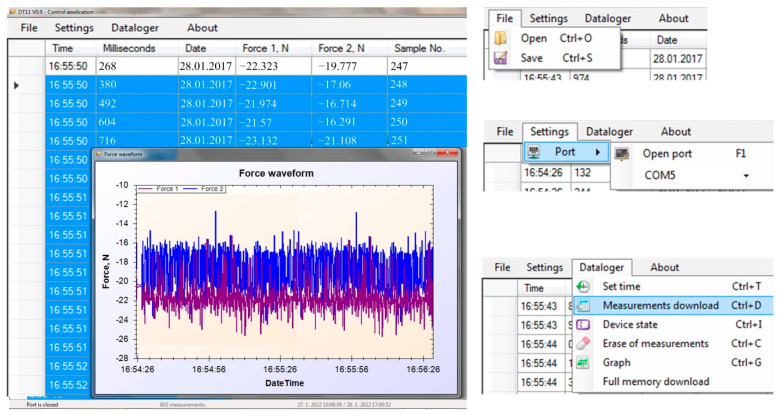
Software for the data acquisition system (the numerical data in the picture are for illustration purposes only).

The proposed software offers the basic options to select the port to connect to the datalogger with PC, or open and save the file with data (Figure 12). The software allows the display of a graph with measured data, where the x-axis is time (hour/minute/second) and the y-axis is force (N). The columns entitled Force 1 and Force 2 represent the data from the load cell within the tire track and outside the tire track, depending on the actual connection of the datalogger to the load cells.

### 3.2. Calibration Test

**Figure 13:**
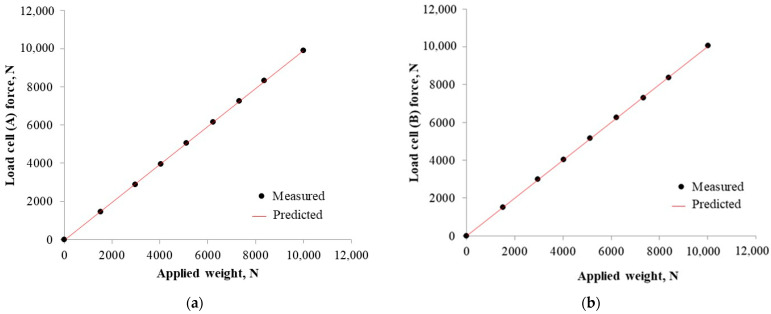
Results of calibration tests relating the output data on the load cell: (**a**) A; (**b**) B.

The calibration test involved applying a series of known forces resulting from the steel discs applied to the load cells. Both load cells, A and B, as shown in Figure 6, were connected in the series. The weights applied to load the cells were in a full measurement range of 0–10,000 N. The datalogger of the measurement system recorded the resulting forces. Graphs were made of the results, comparing the predicted values and applied weights (Figure 13). The load acting on the load cells and the output force showed significant linear correlations within the measurement range (R^2^ = 0.99, *p* < 0.0001). The results of the calibration test confirmed that the applied thrust weight corresponds to the forces recorded by the datalogger, considering the acceptable deviation to be lower than 4%. 

### 3.3. Practical Application under Field Conditions

**Figure 14:**
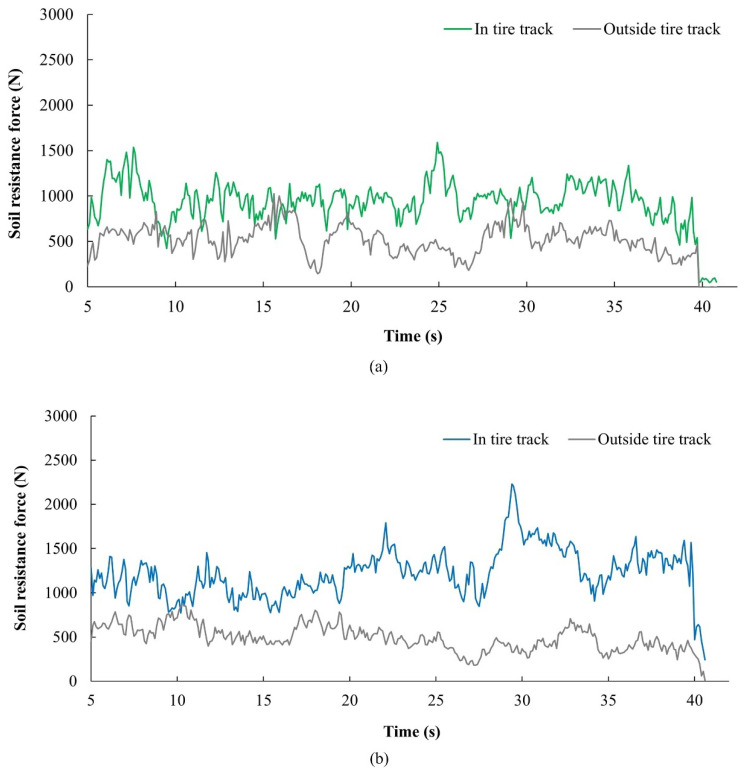

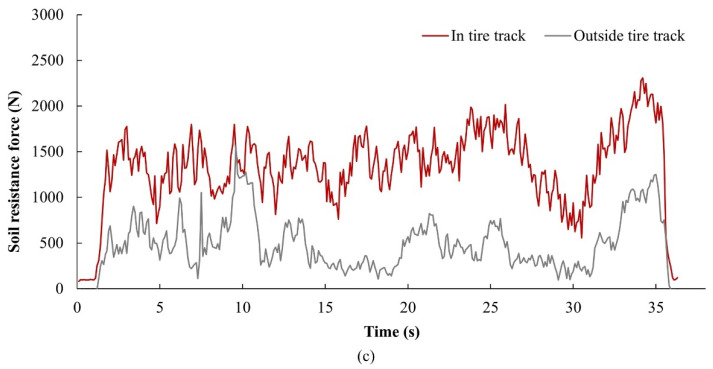
Results of experimental measurements: (**a**) one passage; (**b**) two passages; (**c**) three passages.

The measurement system was tested under field conditions. The experimental measurements of soil resistance forces are shown in Figure 14. The graphs indicate the variation of the measured parameter corresponding to output signals from the load cells. The oscillation of signals results from variation of soil properties. The soil is not ideally homogenous because it is composed of different fractions and may contain stones, roots or other solid parts. 

**Figure 15:**
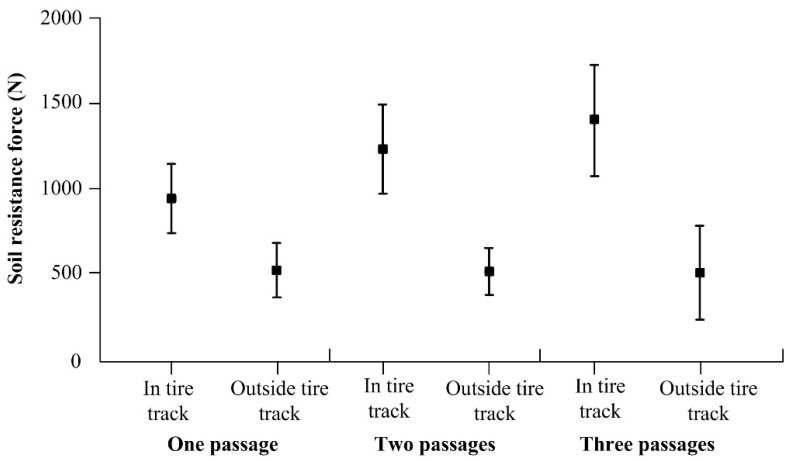
Statistical differences among data measured by the SRSF.

The normality of the measured data was verified by the χ2 test at a significance level of α = 0.05. The test result showing a parameter *p* > 0.05 was considered to be normally distributed data. Otherwise, the normal distribution hypothesis was rejected at the chosen level of significance. The data measured in the tire track after three passages showed deviation from normal distribution according to the χ2 test (*p* > 0.05). Therefore, differences between individual experiments were verified by the non-parametric analysis of variance with Dunn’s significance test at the level α = 0.05. The case *p* ≤ α was considered to be a statistically significant difference. The mean values of 947 N, 1240 N and 1402 N (standard deviation of 202 N, 263 N and 333 N) were calculated from the data measured within the tire track after one, two and three tractor passages. A statistically significant difference between the soil outside the tire track after one passage, and also after one and two passages, was expressed by *p* < 0.0001, and after two and three passages by *p* < 0.05 (Figure 15). The soil resistance forces measured outside the tire track showed similar mean values of 514 N, 516 N and 506 N (standard deviation of 165 N, 134 N and 278 N) in case of one, two and three tractor passages, respectively. The differences among the data measured outside the tire track were insignificant considering *p* > 0.99 (Figure 15). Statisticaly insignicant differences were verified in initial soil propertties after soil cultivation. 

The results showed that soil resistance force increased with the number of tractor passages. The highest increase reached a value of 85% after one passage. The subsequent passages showed lower soil resistance increases of 30% and 13% after two and three passages. The lowest increase in soil resistance force after three passages is also characterized by *p* < 0.05 in comparison with *p* < 0.0001 after one and two passages. The results correspond with the generally known fact that the highest increase in soil compaction is observed after the first tractor passage due to a decrease in soil porosity. The increase is lower after each subsequent passage. 

**Figure 16:**
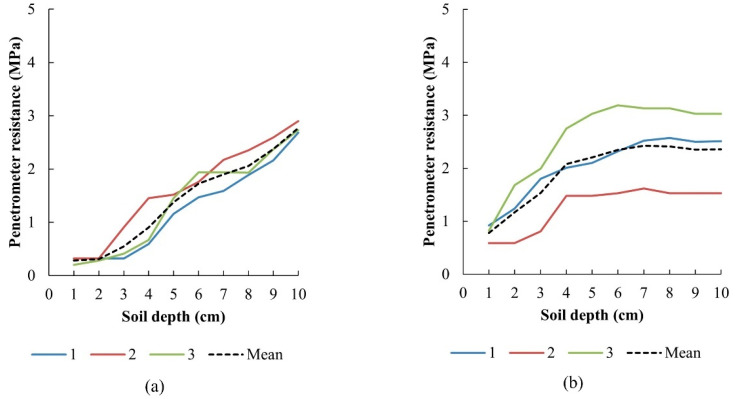

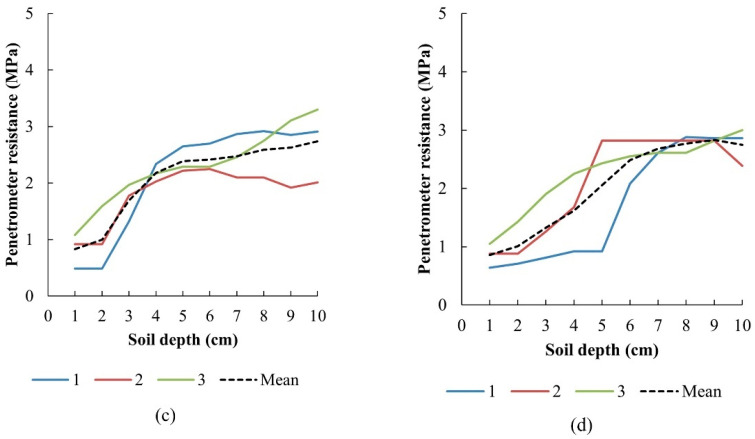
Penetrometer resistance measured three times: (**a**) outside the tire track, (**b**) one passage, (**c**) two passages, (**d**) three passages.

Standard measurements of penetrometer resistance and soil bulk density were performed for comparison with the data recorded by the measurement system. The dependences of penetrometer resistance on the soil depth are shown in Figure 16. The broken line is the mean calculated from data measured three times. 

The penetrologger was recording the penetrometer resistance every 1 cm. The average values of the three measurements are listed in Table 1. The results of penetrometer resistance are characterized by relatively high variation due to there being various soil properties in the small point of penetration (diameter of penetrometer cone) considering low cohesion of the cultivated soil in the surface layers. Soil properties outside the tire track (initial soil properties) are clearly characterized by penetrometer resistances at depths from 1 to 9 cm (Figure 16a and Table 1). The lowest values were measured at the lowest soil depths due to the highest soil porosity in surface layers after cultivation. Values after all tractor passages indicated an increase in soil compaction in surface layers in comparison with the initial soil condition. Typical soil compaction increased with the number of tractor passages and is characterized in soil depths from 6 to 9 cm. Penetrometer resistance increased after each tractor passage. The soil depth of 10 cm is not suitable for soil compaction evaluation because it is near uncultivated soil layers (Figure 16). In [43,44], soil compaction due to tractor traffic measuring of the vertical penetrometer resistance was studied. The mean values of this parameter showed an increase in soil compaction after tractor passes. The experiments showed that the results of penetrometer resistance are affected by human factors when the handheld penetrologger is used and variability of measured values due to variability of soil properties. To obtain precise results when this method is used, practical experiences with evaluation of extreme values are required. On the other hand, the on-the-go sensor with a data acquisition system eliminates measurement errors and generates a data file more suitable for statistical processing and detailed characterization of measured area. 

Soil bulk density is another parameter often used for evaluation of soil compaction. Therefore, it was used for comparison with the measurements of soil resistance force performed by the measurement system. The lowest value of 1.01 g·cm^−3^ was measured outside the tire track (Table 2). Tractor passages caused the increase in this parameter. Soil bulk density reached values of 1.08, 1.13 and 1.25 g·cm^−3^ in the case of one, two and three tractor passages, respectively. An increasing trend of soil bulk density after tractor passages corresponds with an increase of soil resistance force recorded by the proposed measurement system. In [45], it was shown that soil compaction negatively affects the soil physical properties through increasing the soil bulk density. In [46], the effect of machinery passages on soil bulk density was studied. The research showed an increase in soil bulk density due to the passage of two-axle machines. Similar to penetrometer resistance, the evaluation of soil compaction by soil bulk density requires manual human work and considerable time in the case of a large area. A manual sampling process requires practical experience and may cause measurement errors in comparison with the SRSF. 

The research presented in this paper has demonstrated the usefulness of the soil resistance force sensor with the proposed data acquisition system. It allows practical evaluation of soil mechanical properties within and outside the tire track along the field based on the on-the-go principle. The soil properties within the tire track were theoretically and practically researched by many authors [8,10,17,43,44,47]. The pressure acting on the soil can be theoretically calculated from the tire contact area and load acting on the wheel. Therefore, a larger area and lower load is a prerequisite for less soil damage. Besides these basic parameters, many other factors, such as wheel slip, tractor drawbar pull, tractor velocity, soil moisture, agrotechnical operation, etc., affect the interaction between the tractor and soil. To obtain complex information about the influence of agricultural machinery on the soil, the SRSF based on the on-the-go principle is suitable and useful.

## 4. Conclusions

At present, tractor passages through fields are necessary for agricultural production. The original design of the on-the-go soil resistance force sensor (SRFS) evaluating the soil mechanical resistance can be used to characterize soil compaction or traction for tillage tools and has potential for research and practical applications. The main contributions of this study are as follows:

- The design of SRFS is based on two cutting blades which cut through the soil within the tire track and outside the tire track. The design allows the comparison of the uncompacted and compacted soil after passage of agricultural machinery (a tractor was used in our case) in real time. The design allows the setting of the balance of moments acting on the sensor lever mechanism, which simplifies the measured data evaluation.
- The data acquisition system was proposed to convert the mechanical forces acting on the cutting blades into data that can be manipulated by the personal computer. The compact design and simple operation by only two switches and LED indication of the recording process were considered for agricultural operation. The specific software was developed to transfer the data recorded in the datalogger to the computer. The analysis of the recorded signal and the sampling frequency of 10 Hz was verified based on FFT (Fast Fourier transformation).
- The SRFS was tested under field conditions. Soil resistance forces were measured by SRFS with the proposed data acquisition system. The results showed the statistically significant difference between soil mechanical resistances measured outside the tire track and within the tire track after one, two and three tractor passages. The data measured by SRSF showed an increase in soil resistance force after tractor passages. The lowest values were measured outside the tire track and the highest after three tractor passages.
- The data measured with the SRSF were compared with the standard measurements of penetrometer resistance and soil bulk density. The standard methods indicated an increase in soil compaction due to tractor passages at typical variability resulting from their principles. Their main disadvantages are a need for human work which may cause measurement errors and considerable time in the case of large areas.

Based on the results of this study, the on-the-go measurement device with the proposed data acquisition system is suitable for evaluation of soil mechanical resistance and is an innovative technique in comparison with standard methods.

## Figures and Tables

**Table 1:** Penetrometer resistances (mean/standard deviation, MPa).

Place of Measurement	Tractor Passage	Depth (cm)									
		1	2	3	4	5	6	7	8	9	10
outside tire track	0	0.28/ 0.07	0.31/ 0.02	0.55/ 0.31	0.90/ 0.47	1.38/ 0.19	1.72/ 0.05	1.90/ 0.29	2.06/ 0.06	2.37/ 0.21	2.77/ 0.11
within tire track	1	0.78/ 0.16	1.17/ 0.54	1.53/ 0.63	2.08/ 0.64	2.20/ 0.78	2.35/ 0.83	2.42/ 0.75	2.41/ 0.81	2.35/ 0.76	2.36/ 0.76
	2	0.83/ 0.31	1.00/ 0.55	1.69/ 0.34	2.18/ 0.15	2.39/ 0.23	2.41/ 0.24	2.48/ 0.38	2.59/ 0.43	2.63/ 0.62	2.74/ 0.66
	3	0.86/ 0.21	1.01/ 0.37	1.32/ 0.54	1.62/ 0.67	2.06/ 1.11	2.48/ 0.37	2.68/ 0.12	2.77/ 0.14	2.83/ 0.02	2.75/ 0.31

**Table 2:** Data on soil bulk density (g·cm^−3^).

Place of Measurement	Tractor Passage	Measurement Repetition			Mean	Standard Deviation
		1	2	3		
outside tire track	0	0.89	1.01	1.1	1.01	0.11
within tire track	1	1.13	1.09	1.12	1.08	0.02
	2	1.14	1.09	1.14	1.13	0.03
	3	1.33	1.23	1.21	1.25	0.06

## Data Availability

Not applicable.
